# High body mass index and triglyceride levels at health checkups increase the risk of new-onset chronic kidney disease and worsening renal function: the TAMA MED Project-CKD

**DOI:** 10.1007/s10157-024-02507-5

**Published:** 2024-05-20

**Authors:** Tomohiro Kaneko, Eitaro Kodani, Hitomi Fujii, Hiroyuki Nakamura, Hajime Sasabe, Yutaka Tamura

**Affiliations:** 1 Department of Nephrology, Nippon Medical School Tama-Nagayama Hospital, 1-7-1 Nagayama, Tama, Tokyo 206-8512 Japan; 2 Department of Cardiovascular Medicine, Nippon Medical School Tama-Nagayama Hospital, 1-7-1 Nagayama, Tama, Tokyo 206-8512 Japan; 3 TAMA CITY Medical Association, 5-15 Nagayama, Tama, Tokyo 206-0025 Japan; 4 Tama-Center Mirai Clinic, 1-38 Ochiai, Tama, Tokyo 206-0033 Japan

**Keywords:** BMI, Chronic kidney disease, Health checkups, Systolic blood pressure, Triglycerides

## Abstract

**Background:**

Health checkups are important in patients with chronic kidney disease (CKD), which is not easily accompanied by subjective symptoms. CKD can be caused or aggravated by factors that have not yet been identified.

**Methods:**

This retrospective cohort study included 7 483 patients who underwent specific annual health checkups at a medical institution in Tama City, did not have CKD in 2012, and continued to undergo checkups (aged 40–74 years). We examined the risk factors for new-onset CKD and 1.5-fold increase in serum creatinine levels among laboratory values from 2012 to 2020.

**Results:**

Age, body mass index (BMI), triglyceride levels, atrial fibrillation, and medication for hypertension (HT) and diabetes mellitus were independent risk factors for proteinuria, whereas current smoking, BMI, systolic blood pressure (SBP), and medication for HT were independent risk factors for estimated glomerular filtration rate < 60 mL/min/1.73 m^2^. SBP, triglyceride levels and medication for HT were risk factors for a 1.5-fold increase in serum creatinine levels during course of the study. The cut-off values of BMI for eGFR < 60 mL/min/1.73 m^2^ were 22.2 (men 24.7, women 22.1) kg/m^2^ and fasting triglyceride levels for a 1.5-fold increase in serum creatinine level were 171 (men 247, women 170) mg/dL, respectively.

**Conclusions:**

Health checkups provide information to prevent new-onset CKD and worsening of renal function. It is necessary to increase the rate of health checkups and visits to medical institutions after health checkups as well as to use these results for health guidance.

## Introduction

Chronic kidney disease (CKD) is an urgent issue that needs to be addressed as the number of patients requiring dialysis is increasing worldwide. It is a serious risk factor for cardiovascular diseases and has a significant impact on the health of the population [1]. A systematic analysis of the 2017 Global Burden of Disease Study reported that CKD is a highly prevalent condition that contributes to a substantial proportion of the global disease burden; however, over the past 27 years, the burden of CKD has not declined to the same extent as that of many other important non-communicable diseases [2]. Early detection is crucial to reduce CKD; however, many cases of CKD are already advanced at the time of diagnosis because most CKDs worsen without subjective symptoms. Health checkups are of great significance for the early diagnosis of CKD, and the rate of health checkups should increase.

In Japan, specific health checkups, ‘Tokutei kenshin’, intended for individuals who have national health insurance were introduced in 2008 to identify lifestyle-related diseases. Although specific health checkups are useful for the diagnosis of metabolic syndrome, they were initially insufficient to diagnose CKD because the evaluation of the serum creatinine level was removed from the mandatory list. Therefore, in Tama City, in the suburbs of Tokyo, serum creatinine level was included as an essential examination in specific health checkups for the diagnosis of CKD since 2012.

CKD is reportedly caused or exacerbated by various factors [3]. Residual risk factors for new-onset CKD or worsening renal function may be among the test results examined during the health checkups. However, measurement of blood creatinine in specific health checkups is still limited to those whose blood pressure or blood glucose levels meet certain criteria. Risk analysis of CKD using the usual specific health checkups data may result in limitation. In this study, we examined the results of health checkups in Tama City for risk factors for the development of CKD and worsening of renal function during an 8-year study period.

## Materials and methods

### Study design of the TAMA MED Project-CKD

The TAMA MED Project-CKD [4], Project-AF [5], and Project-Frail [6] retrospective cohort studies were conducted to clarify the prevalence and incidence of CKD, atrial fibrillation (AF), and frailty, respectively, in the general population. The study protocol conformed to the Declaration of Helsinki and was approved by the Ethics Committee of our institution (approval number 529, 2017). The participants were enrolled in the National Health Insurance system and underwent consecutive annual specific health examinations at a clinic or hospital belonging to the Tama City Medical Association. All participants were aged 40–74 years at the time of entry because the specific health checkups are open for subjects aged ≥ 40 years, and individuals aged ≥ 75 years are not eligible for this insurance. Age at the end of the fiscal year was recorded in the Tama City database and was used for subsequent analyses. All participants completed the questionnaire, which included items to evaluate self-reported past history, such as cardiac disease, stroke, gastrointestinal disease, renal disease, malignancy, and so on; present illness (the presence of any subjective symptom at the time of specific health checkups, including non-cardiac symptoms); habitual status, including current smoking; and medication use. Body height and weight, systolic blood pressure (SBP), and diastolic blood pressure (DBP) were measured in all participants. In addition, body mass index (BMI) was calculated, and participants were examined for smoking habits and medication status for hypertension, diabetes mellitus, and hyperlipidaemia. Plasma haemoglobin, fasting plasma glucose, glycated haemoglobin (HbA1c), serum low-density lipoprotein (LDL) cholesterol, triglyceride levels, and urine protein and sugar levels were examined using the dipstick test. In addition, a standard 12-lead electrocardiogram was recorded in all participants as a Tama City-specific mandatory optional examination since 2008. Serum creatinine levels were measured in all participants since 2012.

### Statistical analysis

Data are presented as mean ± standard deviation. The statistical significance of differences in means was analysed using the Student’s t-test. Statistical significance was set at P < 0.05 for all analyses. The Cox proportional hazards model was used to investigate the influence of clinical factors. Hazard ratios and 95% confidence intervals (CIs) were calculated for the presence of categorical variables and each 1-unit increase or decrease in continuous variables. We first extracted variables with significant hazard ratios from the univariate analysis. In addition, to detect independent risk factors, a multivariate analysis was conducted using significant variables in the univariate analysis. Kaplan–Meier curves were generated to show the cumulative incidence of new-onset CKD and progressive decline in renal function from 2012 to 2020, and the time to events was compared between BMI, SBP, triglyceride levels, and haemoglobin classes using the log-rank test. The predictive ability of each risk factors and the optimal cutoff values for which interventions would be possible were determined using the time-dependent area under the receiver-operating-characteristic curve (AUC). Statistical analyses were performed using the SPSS software (version 23.0; IBM, Armonk, NY, USA).

## Results

**Fig. 1:**
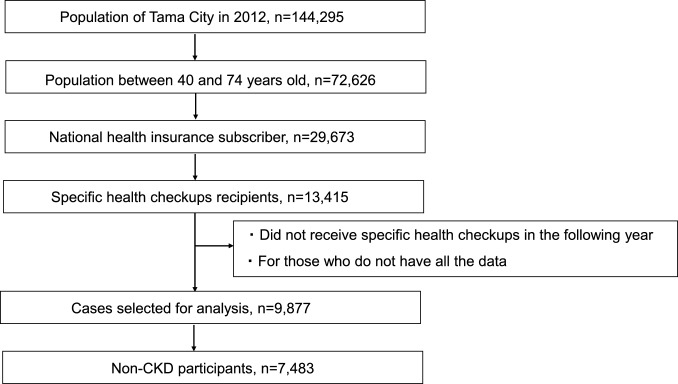
Selection charts of the study population

The population of Tama City in 2012 was 144,295, of which 72,626 were between the ages of 40 and 74. There were 29,673 people with National Health Insurance system, of whom 45.2%, or 13,415 people, underwent the specific health checkups in 2012. Data were obtained from 9877 individuals who underwent medical checkups in 2012 and continued to undergo medical checkups for at least 2 years thereafter (Fig. 1). Of these, 7483 patients with negative proteinuria by dipstick test and an eGFR of ≥ 60 mL/min/1.73 m^2^ were included as non-CKD participants (75.8% of the total). Table 1 shows the characteristics of the participants during the specific health checkups. The age of the participants ranged from 40 to 74 years, with an average age of 65.4 ± 7.2 years. Among the participants, 38.9% were men; there was a considerable difference in the health checkup uptake rate between the sexes.

**Table 1:** Participant characteristics

No. of participants	7483
Age (years)	65.4 ± 7.2
Sex, men	2911 (38.9)
BMI (kg/m^2^)	22.4 ± 3.1
Systolic BP (mmHg)	126.2 ± 16.0
Diastolic BP (mmHg)	74.7 ± 10.2
Current smoker	1495 (20.0)
Blood examinations	
Hb (g/dL)	13.8 ± 1.2
LDL-C (mg/dL)	125.1 ± 29.6
Triglycerides (mg/dL)	110.4 ± 84.2
Glucose (mg/dL)	96.7 ± 18.4
HbA1c (NGSP) (%)	5.2 ± 0.6
Creatinine (mg/dL)	0.7 ± 0.1
eGFR (mL/min/1.73m^2^)	77.5 ± 13.4
Urinalysis	
Urine sugar	179 (2.4)
Electrocardiogram	
AF	60 (0.8)
Medication	
For hypertension	2264 (30.3)
For diabetes mellitus	429 (5.7)
For hyperlipidaemia	1419 (19.0)

Data shown as n (%) or mean ± standard deviation

*BMI* body mass index, *BP* blood pressure, *Hb* haemoglobin, *LDL-C* low-density lipoprotein cholesterol, *HbA1c* glycated haemoglobin, *NGSP* national glycohaemoglobin standardization program, *eGFR* estimated glomerular filtration rate, *AF* atrial fibrillation

### Predictors for new-onset chronic kidney disease

We analysed the risk factors for proteinuria during an 8-year course of health checkups in patients without CKD in 2012. There were 1,701 participants (22.7%) with outcomes during the 8-year period (Table 2). In the univariate analysis, age; BMI; SBP; HbA1c; triglyceride levels; AF; and medications for hypertension, diabetes mellitus, and hyperlipidaemia were associated factors. Among these, age, BMI, triglyceride levels, AF, and medications for hypertension and diabetes mellitus were identified as significant risk factors in the multivariate analysis (Table 3a). Non-significant variables in univariate analysis are not shown in the tables. Age, BMI, triglyceride levels, anaemia, AF, and medications for hypertension for men (Table 3b), and age, BMI, triglyceride levels, and medications for diabetes mellitus for women (Table 3c) were identified as significant risk factors in the multivariate analysis.

**Table 2:** Number of participants and outcomes

	Number of eligible participants	Observational period, years	Number of participants with outcomes (%)
Proteinuria	7483	5 [2, 8]	1701 (22.7%)
eGFR < 60 mL/min/1.73 m^2^	7483	4 [2, 8]	518 (6.9%)
serum creatinine levels being > 1.5 times	7483	7 [2, 8]	115 (1.5%)

Observation periods are median [interquartile range]

*eGFR* estimated glomerular filtration rate

**Table 3:** Significant indicators of new-onset chronic kidney disease (proteinuria) in overall (**a**), men (**b**) and women (**c**) (Cox proportional hazard model)

a. Overall				
Variables (2012 data)	Univariate		Multivariate	
	HR (95% CI)	P value	HR (95% CI)	P value
Age (/1 year increase)	1.062 (1.052–1.072)	< 0.001	1.060 (1.049–1.071)	< 0.001
BMI (/1 kg/m^2^ increase)	1.048 (1.033–1.063)	< 0.001	1.045 (1.027–1.063)	< 0.001
SBP (/10 mmHg increase)	1.065 (1.034–1.098)	< 0.001	0.998 (0.965–1.033)	0.928
HbA1c (/1% increase)	1.090 (1.016–1.169)	0.016	0.909 (0.814–1.015)	0.090
Triglycerides (/10 mg/dL increase)	1.007 (1.004–1.011)	< 0.001	1.009 (1.005–1.014)	< 0.001
AF	2.077 (1.410–3.060)	< 0.001	1.781 (1.187–2.673)	0.005
Medication for hypertension	1.423 (1.292–1.568)	< 0.001	1.181 (1.058–1.319)	0.003
Medication for DM	1.435 (1.201–1713)	< 0.001	1.332 (1.059–1.675)	0.014
Medication for hyperlipidaemia	1.247 (1.116–1.394)	< 0.001	1.027 (0.908–1.162)	0.669

b. Men		
Variables (2012 data)	Multivariate	
	HR (95% CI)	P value
Age	1.052 (1.038–1.066)	< 0.001
BMI	1.036 (1.014–1.059)	0.001
SBP	1.014 (0.971–1.059)	0.534
Triglycerides	1.013 (1.005–1.030)	0.003
AF	1.977 (0.938–4.168)	0.073
Medication for HT	1.133 (0.980–1.311)	0.092
Medication for DM	1.636 (1.168–2.291)	0.004
Medication for HL	1.079 (0.929–1.254)	0.321

c. Women		
Variables (2012 data)	Multivariate	
	HR (95% CI)	P value
Age	1.070 (1.052–1.088)	< 0.001
BMI	1.059 (1.028–1.091)	< 0.001
HbA1c	0.996 (0.8591.155)	0.959
Triglycerides	1.008 (1.003–1.014)	0.003
Hb (/1-g/dL decrease)	0.844 (0.784–0.909)	< 0.001
AF	1.741 (1.069–2.834)	0.026
Medication for HT	1.233 (1.041–1.461)	0.015

Non-significant variables in univariate analysis are not shown

*HR* hazard ratio, *CI* confidence interval, *BMI* body mass index, *SBP* systolic blood pressure, *HbA1c* glycated haemoglobin, *AF* atrial fibrillation, *DM* diabetes mellitus, *HT* hypertension

We also analysed the risk factors for eGFR < 60 mL/min/1.73 m^2^ during the 8-year course of health checkups for those who did not have CKD in 2012. There were 518 participants (6.9%) with outcomes during the 8-year period (Table 2). In the univariate analysis, current smoking, BMI, SBP, HbA1c, triglyceride levels, anaemia, and medications for hypertension and diabetes mellitus were associated factors. Among these, current smoking, BMI, SBP, and medications for hypertension were independently identified as significant risk factors in the multivariate analysis (Table 4a). BMI, SBP and medications for hypertension for men (Table 4b), and BMI for women (Table 4c) were identified as significant risk factors in the multivariate analysis.

**Table 4:** Significant indicators of new-onset chronic kidney disease (estimated glomerular filtration rate < 60 mL/min/1.73 m^2^) in overall (**a**), men (**b**) and women (**c**) (Cox proportional hazard model)

a. Overall				
Variables (2012 data)	Univariate		Multivariate	
	HR (95% CI)	P value	HR (95% CI)	P value
Current smoking	1.459 (1.193–1.785)	< 0.001	0.675 (0.541–0.841)	< 0.001
BMI (/1 kg/m^2^ increase)	1.084 (1.056–1.112)	0.001	1.051 (1.021–1.083)	0.001
SBP (/10 mmHg increase)	1.172 (1.112–1.235)	< 0.001	1.110 (1.048–1.175)	< 0.001
HbA1c (/1% increase)	1.218 (1.101–1.348)	< 0.001	1.057 (0.899–1.243)	0.502
Triglycerides (/10 mg/dL increase)	1.010 (1.006–1.014)	< 0.001	1.005 (0.999–1.011)	0.120
Hb (/1 g/dL decrease)	1.111 (1.034–1.195)	0.004	0.945 (0.866–1.030)	0.198
Medication for hypertension	1.646 (1.382–1.960)	< 0.001	1.322 (1.083–1.598)	0.004
Medication for DM	1.860 (1.386–2.497)	< 0.001	1.369 (0.949–1.973)	0.093

b. Men		
Variables (2012 data)	Multivariate	
	HR (95% CI)	P value
BMI	1.053 (1.014–1.093)	0.007
SBP	1.070 (0.987–1.161)	0.100
Triglycerides	1.014 (0.993–1.036)	0.182
Medication for HT	1.224 (0.930–1.610)	0.149
Medication for DM	1.668 (0.931–2.987)	0.085

c. Women		
Variables (2012 data)	Multivariate	
	HR (95% CI)	P value
BMI	1.069 (1.022–1.118)	0.004
SBP	1.158 (1.067–1.256)	< 0.001
HbA1c	1.136 (0.917–1.407)	0.244
Triglycerides	1.003 (0.996–1.010)	0.392
Medication for HT	1.446 (1.108–1.888)	0.007
Medication for DM	1.187 (0.740–1.904)	0.477

Non-significant variables in univariate analysis are not shown

*HR* hazard ratio, *CI* confidence interval, *BMI* body mass index, *SBP* systolic blood pressure, *HbA1c* glycated haemoglobin, *Hb* haemoglobin, *DM* diabetes mellitus, *HT* hypertension

### Predictors for serum creatinine levels being > 1.5 times

We analysed the risk factors for serum creatinine levels that were 1.5 times higher than the 2012 values during the 8-year course of the study. There were 115 participants (1.5%) with outcomes during the 8-year period (Table 2). In the univariate analysis, BMI, SBP, triglyceride levels, and medication for hypertension were associated factors. Among these, SBP, triglyceride levels, and medication for hypertension were independently identified as significant risk factors in the multivariate analysis (Table 5a). SBP, triglyceride levels and medications for hypertension for men (Table 5b), and SBP for women (Table 5c) were identified as significant risk factors in the multivariate analysis.

**Table 5:** Significant indicators for serum creatinine levels being > 1.5 times (Cox proportional hazard model)

a. Overall				
Variables (2012 data)	Univariate		Multivariate	
	HR (95% CI)	P value	HR (95% CI)	P value
BMI (/1 kg/m^2^ increase)	1.089 (1.032–1.150)	0.002	1.040 (0.981–1.104)	0.189
SBP (/10 mmHg increase)	1.297 (1.165–1.443)	< 0.001	1.244 (1.108–1.396)	< 0.001
Triglycerides (/10 mg/dL increase)	1.013 (1.006–1.019)	< 0.001	1.011 (1.003–1.019)	0.005
Medication for hypertension	2.055 (1.425–2.962)	< 0.001	1.635 (1.117–2.395)	0.012

b. Men		
Variables (2012 data)	Multivariate	
	HR (95% CI)	P value
BMI	1.041 (0.947–1.144)	0.406
SBP	1.217 (1.021–1.451)	0.028
Triglycerides	1.011 (1.003–1.020)	0.008
Medication for HT	2.001 (1.125–3.497)	0.018

c. Women		
Variables (2012 data)	Multivariate	
	HR (95% CI)	P value
BMI	1.037 (0.958–1.122)	0.374
SBP	1.264 (1.083–1.474)	0.003
Medication for HT	1.383 (0.819–2.336)	0.225

Non-significant variables in univariate analysis are not shown

*HR* hazard ratio, *CI* confidence interval, *BMI* body mass index, *SBP* systolic blood pressure, *HT* hypertension

### Influence of systolic blood pressure, body mass index, and triglyceride levels for new-onset chronic kidney disease

**Fig. 2:**
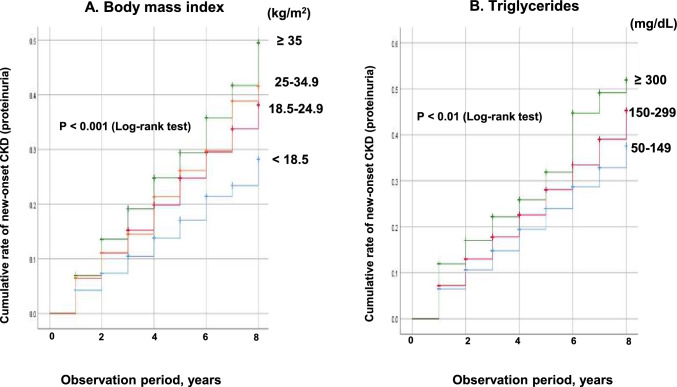
Kaplan–Meier curves for new-onset CKD (proteinuria) between 2012 and 2020 according to (**A**) body mass index (kg/m^2^) and (**B**) triglyceride levels (mg/dL). Analysis for all participants. *CKD* chronic kidney disease

Given that BMI and triglyceride levels were associated with an increased risk of proteinuria in the multivariate Cox hazard model for overall, additional Kaplan–Meier curves were generated to show the cumulative incidence of proteinuria based on BMI and triglyceride classes. There were significant differences in the incidence of CKD (proteinuria) between the presence of a high-value class of BMI (P < 0.001) and triglyceride levels (P < 0.01) in the log-rank test (Fig. 2).

**Fig. 3:**
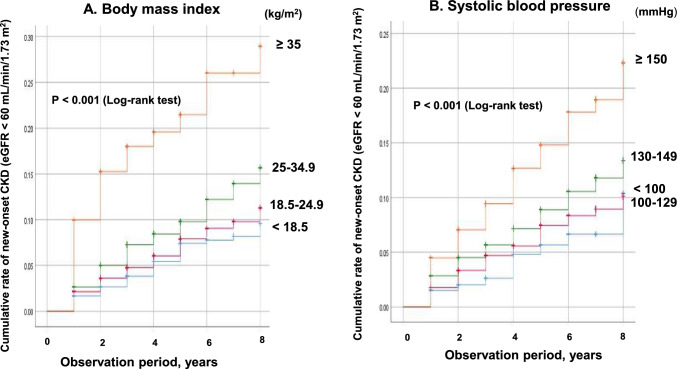
Kaplan–Meier curves for new-onset CKD (eGFR < 60 mL/min/1.73 m^2^) between 2012 and 2020 according to (**A**) body mass index (kg/m^2^) and (**B**) systolic blood pressure (mmHg). Analysis for all participants. *CKD* chronic kidney disease, *eGFR* estimated glomerular filtration rate

BMI and SBP were associated with an increased risk of GFR < 60 mL/min/1.73 m^2^ in the multivariate Cox hazard model for overall. There were significant differences in the incidence of CKD (eGFR < 60 mL/min/1.73 m^2^) between the presence of a high-value class of BMI (P < 0.001) and SBP (P < 0.001) based on the log-rank test (Fig. 3). As for SBP, no difference in risk was observed between SBP was < 100 mmHg and SBP 100–129 mmHg. (Fig. 3B).

The AUC of and the cut-off value of BMI for eGFR < 60 mL/min/1.73 m^2^ were 0.564 and 22.2 kg/m^2^ (0.559 and 24.7 kg/m^2^ for men, 0.543 and 22.1 kg/m^2^ for women, respectively).

### Influence of systolic blood pressure and triglyceride levels for serum creatinine levels being > 1.5 times

**Fig. 4:**
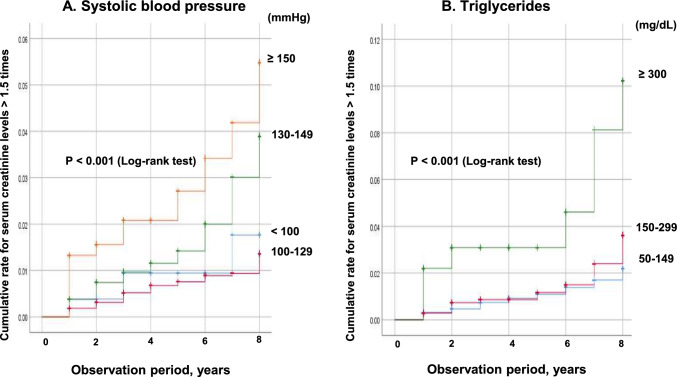
Kaplan–Meier curves for serum creatinine levels being > 1.5 times between 2012 and 2020 according to (**A**) systolic blood pressure (mmHg) and (**B**) triglycerides (mg/dL). Analysis for all participants

SBP and triglyceride levels were associated with an increased risk of serum creatinine levels being > 1.5 times in the multivariate Cox hazard model for overall. There were significant differences in the incidence of worsening of renal function between the presence of a high-value class of SBP (P < 0.001), and triglyceride levels (P < 0.001) based on the log-rank test (Fig. 4). As for SBP, the risk tended to increase when the SBP was < 100 mmHg compared with when the SBP was 100–129 mmHg (Fig. 4A).

The AUC of and the cut-off value of triglyceride levels for a 1.5-fold increase in serum creatinine levels were 0.586 and 171 mg/dL (0.615 and 247 mg/dL for men, 0.552 and 170 mg/dL for women, respectively).

## Discussion

We had hypothesized that residual risk factors for new-onset CKD or worsening of renal function may be among the test results examined during the health checkups. In this study, we used the results of specific annual health checkups in Tama City, Tokyo, to analyse the factors that cause new-onset CKD and worsening of renal function over a period of 8 years. We found that age, BMI, triglyceride levels, and AF were risk factors for new-onset proteinuria, while BMI and SBP were risk factors for reduced renal function. Furthermore, SBP and triglyceride levels were identified as significant risk factors for increasing serum creatinine levels > 1.5 times. High BMI and triglyceride levels at health checkups were suggested to increase the risk of new-onset CKD and worsening renal function.

### Prevalence of chronic kidney disease

As of 1 January 2012, the population of Tama City in Tokyo was 144,295. Specific annual health checkups were available for people aged 40–74 years, and only those with national health insurance were included in this study. The reason that the 9,877 people for whom data were available for this study represent only about 6.8% of the Tama City population may be that many people in this age group have other insurance interventions and that the health checkup uptake rate is less than half (45.8% in 2013). The incidence of CKD among these participants was 24.2%.

### Body mass index as a risk factor for new-onset chronic kidney disease (both proteinuria and eGFR < 60 mL/min/1.73 m.^2^)

In this study, high BMI was found to be an independent risk factor for new-onset CKD (both proteinuria and eGFR < 60 mL/min/1.73 m^2^). Among populations without type 2 diabetes mellitus or hypertension, the incidence of CKD was three times higher in the group with juvenile obesity than in the group without obesity [7]. A study of 320,252 Kaiser Permanente insured persons in the United States showed that BMI ≥ 25.0 kg/m^2^ was a significant risk factor for developing end-stage kidney disease, even after adjusting for smoking, hypertension, and diabetes mellitus [8].

Obesity increases systemic fluid volume, renal plasma flow, and GFR [9], and the mechanism is thought to involve activation of the sympathetic nervous system, increased salt sensitivity in the renal tubules, and increased reabsorption of salt linked to the reabsorption of sugar excreted in the urine due to overeating [10]. Obesity-related glomerular hyperfiltration is ameliorated after weight loss [9]. The expression of leptin and resistin is increased in obesity. Hyperleptinemia is associated with the onset of CKD, and sympathetic nervous system activation and sodium reabsorption-promoting action in the kidney cause an increase in blood pressure [11].

The proportion of adults with obesity has significantly increased worldwide [12]. The significance of obesity in new-onset and progression of CKD is expected to increase, and preventive medicinal approaches are crucial. In this study, the BMI cut-off value for new-onset CKD (eGFR < 60 mL/min/1.73 m^2^) was 22.2 kg/m^2^ (24.7 kg/m^2^ for men, 22.1 kg/m^2^ for women). We believe that this is a target value to prevent the deterioration of renal function for Japanese of this age group.

### Triglyceride level as a risk factor for proteinuria and worsening of renal function

In recent years, hypertriglyceridemia has been reported to be associated with onset and progression of various diseases. In a case–control study of 1891 patients with type 2 diabetes mellitus and nephropathy and 3683 patients with type 2 diabetes mellitus without nephropathy or retinopathy matched for background factors, high triglyceride and low high-density lipoprotein cholesterol levels were risk factors for new-onset nephropathy [13]. A Japanese cohort study that followed 102,900 patients without CKD for 2 years reported that a high triglyceride/high-density lipoprotein cholesterol ratio was associated with new-onset CKD and a decrease in eGFR [14].

Therefore, controlling triglyceride levels is important for the suppression of CKD progression. In our study, the target fasting triglyceride level to avoid worsening renal function was 171 mg/dL (247 mg/dL for men, 170 mg/dL for women) in the fasting state. In our study, we found a dissociation between men and women in the cutoff values for triglycerides. An analysis of big medical data of 560,000 people aged 18–74 years in Japan reported that the optimal triglyceride levels to predict cardiovascular disease were 130 mg/dL and 90 mg/dL for men and women, respectively [15]. Similar to our study targeting CKD, it is suggested that more stringent standards for triglycerides are needed in women. In recent years, drugs with new mechanisms to lower triglyceride levels have been used clinically one after another, and evidence for their effects on CKD is expected to develop.

### Hypertension as a risk factor for eGFR < 60 mL/min/1.73 m^2^ and worsening of renal function

The rate of decrease in GFR has been reported to be 4–8 mL/min/1.73 m^2^/year in patients with comorbid hypertension (0.3 mL/min/1.73 m^2^/year in patients without hypertension) [16]. Our results once again demonstrate that CKD and hypertension are closely related to each other, and that hypertension causes and worsens CKD. In our study, systolic blood pressure < 130 mmHg showed a reduced risk for new onset CKD (eGFR < 60 mL/min/1.73 m^2**)**^ and serum creatinine levels > 1.5 times. However, there was a tendency for the risk to increase if the systolic blood pressure was below 100 mmHg, and it was thought that caution should be taken to avoid excessively lowering blood pressure. According to the Third National Health and Nutrition Examination Survey, a detailed study of the relationship between CKD and blood pressure and age revealed that there is a J-curve phenomenon between blood pressure and CKD incidence in older patients aged ≥ 60 years, with or without hypertension treatment [17]. In addition, in the Gonryo study of 2655 Japanese patients with CKD (mean age, 60 years), SBP < 110 mmHg and DBP < 70 mmHg were found to be independent risk factors for cardiovascular events and all-cause mortality. Low blood pressure was not observed as a significant risk factor for progression to end-stage kidney disease [18].

### Other risk factors for new-onset chronic kidney disease

AF was an independent risk factor for new-onset proteinuria in non-CKD participants in men. In our previous report, AF was found to be a risk factor for the appearance of proteinuria, even over a short period of 2 years [19]. We previously reported that CKD classification is significantly associated with new-onset AF in the general population [20]. AF may cause CKD and vice-versa.

### Study limitations

The present study had several limitations. First, the participants were limited by age and insurance coverage as individuals with social insurance and/or aged < 40 or ≥ 75 years at disease onset were not eligible for these specific health checkups. Second, the diagnosis of CKD was based solely on the results of a single health checkup. CKD was defined as having aberrant test results over a period of ≥ 3 months, and this may have resulted in overestimating the number of CKD-diagnosed participants. Third, the uric acid level was not measured in this study, even though it is a risk factor for CKD, as it was not compulsory to test it in specific health checkups. However, recognizing its significance, Tama City added it as a compulsory test from the start of the 2016 financial year. Therefore, we were unable to add this to the current analysis; however, this should be considered in future analyses to ensure better estimates.

## Conclusion

High BMI and triglyceride levels were risk factors for new-onset CKD and worsening of renal function. The cut-off values of BMI for eGFR < 60 mL/min/1.73 m^2^ were 22.2 (men 24.7, women 22.1) kg/m^2^ and fasting triglyceride levels for a 1.5-fold increase in serum creatinine level were 171 (men 247, women 170) mg/dL, respectively.

To prevent the development of CKD, it is necessary to increase the rate of health checkups and visits to medical institutions after health checkups as well as to use these results for health guidance and general medical care.

## Data Availability

No new data were generated or analysed in support of this research.
